# The impact of transparency and imitation over complex networks in strategic classification

**DOI:** 10.1371/journal.pone.0346241

**Published:** 2026-07-10

**Authors:** Flavia Barsotti, Fernando P. Santos

**Affiliations:** 1 ING Analytics, ING Bank N.V., Amsterdam, The Netherlands; 2 Delft Institute of Applied Mathematics, TU Delft, The Netherlands; 3 Informatics Institute, University of Amsterdam, Amsterdam, The Netherlands; Teesside University, UNITED KINGDOM OF GREAT BRITAIN AND NORTHERN IRELAND

## Abstract

Classification algorithms are widely used in critical domains such as healthcare, bank loans, credit and fraud detection. These systems should be transparent, yet it remains unclear how individuals will use explanations to adjust their own features. Individuals often access multiple sources of information, from insights provided by institutions to experiences shared among peers. Based on the information received, individuals may decide to strategically adapt to obtain a favourable outcome, honestly improving or attempting to game the system. This paper studies the impact of transparency and social information on strategic classification. We assume that agents adapt based on best response and behavioural imitation along the edges of social networks. We observe that increasingly opaque decision rules can negatively impact the utility of institutions, especially in dense social networks. The number of False Positives is reduced in networks with a lower average degree, when users imitate the average behaviour, as opposed to the most extreme behaviours. When imitating the most extreme behaviour among their connections, users change their features to a large extent in networks with a higher average degree (i.e., higher density). This applies to both honest improving and gaming, with more pronounced impacts in the case of the latter, creating an additional source of risk for institutions. Our model and results reveal that behavioural imitation patterns and social network effects influence the downstream effects of algorithmic transparency.

## 1 Introduction

Classification algorithms are widely used in critical domains that severely impact individuals’ well-being [1]. As banking and finance increasingly rely on artificial intelligence (AI) and machine learning (ML) algorithms to make high-stakes decisions, for example approving loans, setting credit limits, or detecting fraud, concerns about algorithmic transparency and strategic classification became prominent [2]. Many of these models operate as ’black boxes’, leaving consumers, regulators, and even developers uncertain about how specific decisions are made. This opacity can erode trust, perpetuate biases, and hinder accountability. Offering transparent algorithms and *actionable recourse* is desirable and in some cases legally mandatory [2–5]. How will individuals adapt to increasingly transparent algorithms remains an open question [6,7].

Transparency may create new opportunities for users but also new challenges and risks. As individuals become aware of algorithms’ inner workings, they may honestly adapt to be classified as positive. This is desirable. However, they can also alter their behaviour strategically to obtain favourable outcomes, thereby degrading the performance and fairness of the system [6,8,9]. When individuals alter their features, they may create *data distribution shifts* that require algorithm retraining and even render previous explanations misleading [10]. As a consequence, strategic adaptation might introduce a fundamental mismatch between training and testing data. Algorithmic transparency should be paired with models that remain robust under *data shifts* [11] due to users’ adaptation, to ensure that *information knowledge* does not compromise reliability or exacerbate vulnerabilities. This goal has motivated works on **strategic classification**, aiming at developing algorithms that remain robust after strategic adaptation [7,12–15].

The challenge of dissuading strategic behaviour is particularly acute in the finance sector, where algorithmic decision making in credit provision is prevalent and fraud is a risk. Previous works have identified examples of information manipulation in loan applications [16,17], including when algorithmic rules become transparent [18]. In fact, first-party fraud, which includes intentionally misrepresenting one’s intentions or financial situation, was recently highlighted as the leading source of fraud in digital transactions [19]. More recently, methods based on Generative AI (GenAI) emerged as a crucial ingredient in providing misleading information. Examples include using Generative AI to fabricate documents such as pay stubs, bank statements, or tax forms that appear authentic to fraud detection systems. Tools like ChatGPT or image generation models (e.g., DALLE or Midjourney) are able to produce realistic (yet fake) financial documents that bypass basic verification [20]. In this context, detection mechanisms must be implemented and regularly calibrated to account for additional sources of manipulation risk.

An important aspect of gaming behaviours (i.e., faking) is the social embedding of individuals being subject to algorithmic decisions. People do not act as isolated individuals deciding rationally [21]. Instead, they also adapt their behaviour through social learning and are subject to social network effects [22,23]. People often rely on information from their peers to form beliefs and make financial decisions [24]. Social learning and peer effects are known to arise even in contexts involving the violation of social norms [25], making it essential to account for these dynamics when analysing potential risks of algorithmic manipulation. Previous works also consider imitation and information sharing in the context of strategic classification [7,26]: in these studies, individuals share the information each other hold about the algorithms’ decision rules. Individuals are still assumed to be fully rational: they first construct an estimate for the classification threshold (i.e., deployed rule), and then adapt through empirical risk minimization. The role of behavioural imitation along social networks remains under-explored, especially in settings where individuals directly imitate the behaviour of others.

From this perspective, the present study examines how transparency in algorithmic decision making influences strategic classification outcomes through social learning and imitation effects within complex networks. Our goal is understanding how individuals adapt when classification algorithms are transparent and how such adaptations may propagate socially. Importantly, the focus here is on the *mechanism and behavioural dynamics* that arise in strategic environments, with the aim of informing safe, equitable, and well designed classification systems.

**Fig 1:**
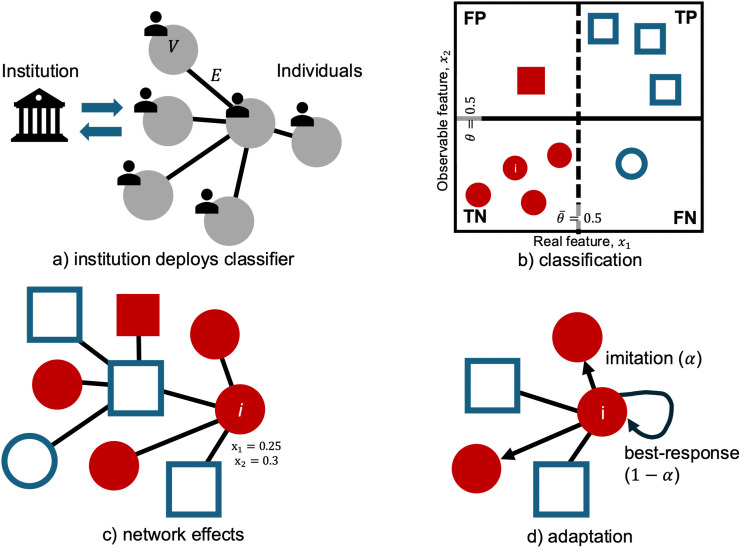
Complex networks and strategic classification. Panel **a)**: We consider a population of *N Individuals* classified by one *Institution*. Individuals are represented as a set of nodes (set *V*: *N* = |*V*|) of a complex network and connected via a set of edges (*E*). Panel **b)**: The Institution aims at accurately classifying individuals in order to provide a service (e.g., concede a loan). Classification is based on the observable feature *x*_2_ (e.g., income submitted with applying to the loan) and actual success is based on the real feature *x*_1_ (e.g., real income). Full red (hollow blue) nodes are the ones not able to repay (able to repay) based on their real feature *x*_1_. Squares are classified as positive by the Institution and circles are classified as negative, based on their observable feature *x*_2_. The latter are the nodes that can decide to adapt. Individuals can adapt their real feature (**improving**) or just their observable feature (**gaming**). As illustrative example, we report a case with classification threshold based on real feature $\bar{\theta }=0.5$ and classification threshold based on observable feature θ=0.5. This enables to detect False Positive (*FP*), True Positive (*TP*), False Negative (*FN*), True Negative (*TN*). Panel **c)**: We assume that adapting nodes, when considering imitation, will take into account the behaviour of neighbours in a social network. As shown in Panel **b)**, Individual *i* results classified as True Negative (*TN*) given $x_{1}=x_{1}(i,t)=0.25$ and $x_{2}=x_{2}(i,t)=0.3$. Panel **d)**: We model one adaptation step: individuals adapt through a combination of best response (with weight 1−α) and behavioural imitation (with weight α).

This paper proposes a mathematical framework to study strategic classification in the presence of behavioural imitation through social networks. Similar to [27,28], and as represented in Fig 1, we focus on the interplay between the *feedback* shared by an Institution (e.g., a bank) and the strategic adaptation of Individuals subject to a generic classification model. Crucially, we assume that individuals influence each other along their social network connections: Individuals previously classified as negative can exchange information on adaptation strategies when re-applying to a service.

We use a binary classification model and study the effects of feedback, imitation and detection in countering faking behaviours via an illustrative example. We introduce a measure to evaluate the average adaptation effort required by Individuals as an indicator of *social burden* effects. We focus on: i) the role of noise in feedback/explanations provided by the Institution, ii) adaptation through imitation of behaviours within a population, iii) the role of social network topology on the risks resulting from strategic classification.

We observe that opaque decision thresholds can negatively impact the institutions’ utility, especially in denser social networks. In this setting, the number of new False Positives (referred to as ΔFP) is reduced in networks with lower average degree (i.e., average number of connections per node). This effect occurs when users imitate the average behaviour in their social networks, as opposed to the most extreme behaviours. We can also observe that users’ change their features to a large extent in networks with a smaller degree, when imitating the average behaviour. The opposite trend is observed when imitating the maximum behaviour.

The paper is organized as follows. Section 2 introduces the mathematical model and our experimental setup, Section 3 presents and discusses the results. Finally, Section 4 provides concluding remarks. The S1 Appendix provides the pseudo-code underlying the experimental setup [29]. Code and data to replicate the results are available from a dedicated repository [30].

## 2 Methods

This Section introduces the mathematical model and the experimental setup in order to provide a formal methodological overview. Let us start by summarizing the key steps of our analysis and methodology: a) We start by assuming that Individuals are classified by Institutions and are characterized by two features, i.e., an *observable feature* and a *real feature* (Section 2.1); b) Classification is based on the observable feature and real success is based on the real feature; the mismatch between the two can negativelly impact the classifier accuracy (Section 2.2); c) After applying the classification algorithms, Institutions provide feedback to the population of Individuals (Section 2.3); d) When adapting, e.g., when deciding which feature to modify, Individuals can imitate their neighbours in a social network (Section 2.4). e) Our experiments aim at measuring how algorithmic accuracy is affected given users adaptation through social learning (Section 2.5).

### 2.1 Institution and population of Individuals

Let us consider a setting with two types of agents: **Institutions** and **Individuals**. We assume one Institution and a finite population of *N* Individuals subject to classification. Fig 1a provides a visual representation of the interaction between one Institution and multiple Individuals that could be part of a social network. The Institution aims at accurately classifying Individuals in order to provide a certain service. In practice, this could be applied in different contexts, from a college deciding to admit a student, to a bank deciding to offer a loan based on the customer creditworthiness. We take the case of credit lending. We assume that the Institution builds a classification algorithm in order to assign a score to each client to determine the final decision of granting or not credit. The transparency level of the feedback shared by the Institution with Individuals is modelled in our setting via parameter σ (see Eq. (7) for more details).

At time *t*, a generic Individual *i* is characterized by a (normalized) **real** feature $x_{1}(i,t)\in [0,1]$, directly causing individuals success (i.e., actually repaying a loan), and an **observable** feature value $x_{2}(i,t)\in [0,1]$, which is used by the Institution to classify users. We recall that, in real world cases, *x*_1_ and *x*_2_ do not necessarily match for all Individuals as declared features might be misreported.

### 2.2 Classification task

Let $\bar{\theta }\in [0,1]$ be the real *success threshold* determining an Individual’s real *success*, e.g., capturing the *real ability* to repay a loan in the credit lending application analysed in this paper. This means that we can state what follows, ∀t:

- when $x_{1}(i,t)\geq \bar{\theta }$, Individual *i* is *successful* (e.g., is able to successfully repay a loan),
- when $x_{1}(i,t)<\bar{\theta }$, Individual *i* is *not successful* (e.g., is not able to successfully repay a loan).

The algorithm deployed by the institution aims at classifying individuals as Positive or Negative to offer them a service (e.g., credit or loan). Let us denote with $\rho_{i}(x_{1}(i,t),\bar{\theta })\in [0,1]$ the *probability of success* of Individual *i* at time *t* which depends on the real feature *x*_1_(*i*,*t*) and *t*he real success threshold $\bar{\theta }$ as follows


$$\begin{array}{c} \rho_{i}(x_{1}(i,t),\bar{\theta })=\left\{ \begin{array}{cc} \frac{1}{1+e^{1/\epsilon (\bar{\theta }-x_{1}(i,t))}}, & \text{if }\epsilon >0\\ H(x_{1}(i,t)-\bar{\theta }), & \text{if }\epsilon =0, \end{array}\right. \end{array}$$ (1)

with $\epsilon \in {\mathbb{R}}_{0}^{+}$ capturing the *noise* between the real feature and the probability of success, and function H(·) being the Heaviside step function


$$\begin{array}{c} H(x)=\left\{ \begin{array}{cc} 1, & \text{if }x\geq 0\\ 0, & \text{if }x<0. \end{array}\right. \end{array}$$ (2)

Function $\rho_{i}(x_{1}(i,t),\bar{\theta })$ is also referred to as the *true* or *target* classifier in [12]. Parameter $\epsilon \in {\mathbb{R}}_{0}^{+}$ introduces a potential disturbance between the real feature *x*_1_ and the probability of success, thus linked with the possibility of predicting whether someone will to repay a loan. In this sense, ϵ represents *classification noise* and allows us to control for the complexity of the classification task faced by institutions.

Based on the level of classification noise ϵ, we have:


$$\begin{array}{c} \forall i,\;\lim_{\epsilon \to \infty }\rho_{\epsilon }(x_{1}(i,t),\bar{\theta })=0.5, \end{array}$$ (3)

meaning that, for all Individuals *i*, irrespective of their real feature *x*_1_(*i*,*t*), as ϵ becomes very large (ϵ→∞), the probability of being a positive example (i.e., repaying a loan) gets closer to 50%. This represents a random scenario.

On the contrary, when ϵ→0 we observe a split, based on the value of the real feature w.r.t. the real success threshold $\bar{\theta }$. In particular:

- i) all individuals *i* with real feature $x_{1}(i,t)\geq \bar{\theta }$ will successfully repay the loan:

$$\begin{array}{c} \forall i:x_{1}(i,t)\geq \bar{\theta },\;\lim_{\epsilon \to 0}\rho_{\epsilon }(x_{1}(i,t),\bar{\theta })=1, \end{array}$$ (4)

- ii) all Individuals with $x_{1}(i,t)<\bar{\theta }$ will be negative examples, i.e., associated to a null probability of success

$$\begin{array}{c} \forall i:x_{1}(i,t)<\bar{\theta },\;\lim_{\epsilon \to 0}\rho_{\epsilon }(x_{1}(i,t),\bar{\theta })=0. \end{array}$$ (5)

The Institution runs the internal classification model and sets a threshold θ∈[0,1], where θ is a parameter learned through methods such as empirical risk minimization. This parameter defines a decision boundary on the observable feature *x*_2_, self-reported by each Individual, which determines a binary classification outcome $\Theta_{i}\in \{0,1\}$, given by


$$\begin{array}{c} \Theta_{i}(x_{2}(i,t);\theta )=\left\{ \begin{array}{cc} 1, & \text{if }x_{2}(i,t)\geq \theta \\ 0, & \text{if }x_{2}(i,t)<\theta . \end{array}\right. \end{array}$$ (6)

Observe that, while $\bar{\theta }$ identifies the real ability to repay a loan based on *x*_1_, the threshold θ determines the classification outcome on the loan application *only* based on the information self-reported by Individuals *x*_2_.

In practice, Institutions may have different classification thresholds θ, and decide to set higher or lower ones depending on how strict they wish to be in terms of requirements on the application process. A lower threshold can be translated into potential acceptance of lower quality applications, while a high threshold would imply potential higher standards set to receive a favourable outcome from the classification.

For each Individual, based on the real probability of success, $\rho (x_{1},\bar{\theta })$, and inferred success based on $\Theta (x_{2},\theta )$, the classification task can have one of four outcomes: False Positive (*FP*), False Negative (*FN*), True Positive (*TP*), True Negative (*TN*). Fig 1b provides an overview of these outcomes.

### 2.3 Feedback and adaptation

A generic Individual *i* infers the classification threshold $\theta_{i}$ used by the Institution, based on the feedback provided by the latter, as


$$\begin{array}{c} {\hat{\theta }}_{i}=\max(x_{2}(i,t),N\sim (\theta ,\sigma )), \end{array}$$ (7)

where ${\hat{\theta }}_{i}$ denotes the estimate of classification threshold $\theta_{i}$ by Individual *i*, *x*_2_(*i*,*t*) is the observable feature self-reported by Individual *i* and N~(θ,σ) represents a value sampled from a normal distribution with mean θ and s*t*andard deviation σ. Parameter σ controls for the accuracy of the feedback provided by the Institution to Individuals.

We tune the transparency level of the feedback provided by the Institution through parameter (σ). In particular, σ captures the uncertainty associated to the knowledge – for Individuals – of the real decision threshold θ set by the Institution on the observable feature *x*_2_. Parameter σ can be interpreted as a measure of *noise* around the real value of the threshold, capturing *information uncertainty* as representative of different levels of *transparency*. In this sense, we also refer to it as level of *feedback noise*. This enables us to create and assess scenarios ranging from *full transparency* (i.e., Individuals have full information about the exact position of the Institution decision boundary, σ=0) to *obscurity* (i.e., individuals can only guess where the decision boundary lies, σ=1). This setting builds on the model proposed in [27,28] and considers a similar conceptual framework to describe the interaction between Individuals and the Institution.

Notice that, intuitively, a high value of σ means that Individuals cannot do better than randomly guessing the threshold used by the Institution. On the contrary, a low value of σ implies that all Individuals have the exact information about the real threshold θ and consider strategic adaptation accordingly.

#### 2.3.1 Adaptation.

Once information is provided, individuals build their estimate of the threshold used by the Institution, ${\hat{\theta }}_{i}$ in Eq. (7), and act based on their adaptation decisions. Let us indicate the amount of fake information provided at time *t* by Individual *i* with


$$\begin{array}{c} f(i,t)=x_{2}(i,t)-x_{1}(i,t),\;f(i,t)\in [0,1], \end{array}$$ (8)

measuring the difference between the observable feature and the real feature for the same individual. We denote the *probability of detection* for Individual *i* at time *t* with *d*[*f*(*i*,*t*)], given by


$$\begin{array}{c} d[f(i,t)]=f(i,t{)}^{1/\phi },\;\phi \geq 0. \end{array}$$ (9)

Since f(i,t)∈[0,1], we introduce parameter ϕ to measure *detection effectiveness*. When ϕ→0 detection always fails; when ϕ=1, we assume a linear dependence of the detection probability on the amount of fake information; when ϕ→+∞, detection never fails and faking is always identified.

Individuals adaptation is not free. To mimic real situations, we assume that individuals must exert an effort to adapt their features. Let us denote with $c_{\iota },c_{f}$, respectively, the cost of truthfully improving and faking regarding the information self-reported by Individuals to Institutions; $c_{d}$ denotes the cost for an Individual of being detected when faking. To assess the impacts of the different costs, we formally define the cost functions as follows:


$$\begin{array}{c} c_{f}=\gamma \cdot c_{\iota },\;c_{d}=(1+\gamma )\cdot c_{\iota },\;\gamma \in [0,1],c_{\iota }\geq 0. \end{array}$$ (10)

In practice, the cost of faking, $c_{f}$, results from, e.g., the effort of cheating on an exam, committing plagiarism or declaring non-existent financial information on income. In general, without considering detection, it is clear that faking in providing information involves a cost naturally much lower than the cost of honestly improving. Taking into account controls put in place by the Institution, detection plays a key role. In this sense, the cost of being detected, $c_{d}$, is imputed when an Individual fakes and the misreported information is detected, e.g., resulting in suspension from college, a fine or an audit. In principle, costs can vary independently. However, parameter γ helps exploring different reasonable combinations of cost values. By tuning γ we can interpolate between extreme scenarios in terms of complexity faced by the Institution: i) for γ=0, Individuals are unlikely to improve (e.g., assuming faking cheap and low detection costs) and ii) for γ=1, Individuals are likely to improve (e.g., assuming faking as expensive as truthful improvement and high detection costs). As main baseline case for the analysis, we consider an intermediate scenario in which γ=0.5, in order to explore the core impacts deriving from transparency and behavioural imitation on complex networks without having extreme cases for the costs.

Let us consider the set of Individuals *i* who received a negative classification outcome. For each Individual *i* in this set, let us consider the estimate about the decision threshold ${\hat{\theta }}_{i}$ in Eq. (7) and the detection mechanism with probability of detection *d*[*f*(*i*,*t*)] in Eq. (9). Once received the feedback from the Insti*t*ution, these individuals define at time *t* + 1 the new pair of features, poten*t*ially different from the original one, depending on their adaptation decision and strategy. We assume that individuals decide the vector of features at time *t* + 1, namely $\vec{x}(i,t+1)=(x_{1}(i,t+1),x_{2}(i,t+1))$, by maximizing the (expected) utili*t*y function *u*(*i*,*t* + 1), given as:


$$\begin{array}{c} \begin{array}{cc} u( & i,t+1)=(1-d[f(i,t+1)]){\hat{\Theta }}_{i}(x_{2}(i,t+1);{\hat{\theta }}_{i})b\\ & -\Delta_{1}(i,t+1)c_{\iota }-f(i,t+1)c_{f}-d[f(i,t+1)]c_{d},\;b\geq 0 \end{array} \end{array}$$ (11)

with $\Delta_{1}(i,t+1)$ indicating the variation in the information regarding the real feature


$$\begin{array}{c} \Delta_{1}(i,t+1):=(x_{1}(i,t+1)-x_{1}(i,t)). \end{array}$$ (12)

Observe that $\Theta_{i}(x_{2}(i,t);\theta )$ represents the output of the binary classification model deployed by the Institution given in Eq. (6); ${\hat{\Theta }}_{i}(x_{2}(i,t+1);{\hat{\theta }}_{i})$ indicates the estimate on the *expected* classification done by Individual *i*; *f*(*i*,*t* + 1) is given in Eq. (8) and *d*[*f*(*i*,*t* + 1)] in Eq. (9). Parameter b≥0 indicates the benefit of receiving a positive classification, i.e., Θ(·)=1, while $c_{\iota },c_{f}$, deno*t*e, respec*t*ively, the cost of improving or faking and $c_{d}$ the cost incurred after being detected given in Eq. (10).

Each term of the expected utility function can be explained as follows. We assume that individuals receive a benefit *b* if they are classified as positive and are not detected to be faking, $(1-d[f(i,t+1)])\cdot {\hat{\Theta }}_{i}(x_{2}(i,t+1);{\hat{\theta }}_{i})\cdot b$; individuals pay an improvement cost $c_{\iota }$ for each unit of improvement in the information regarding their real feature, $\Delta_{1}(i,t+1)\cdot c_{\iota }$; individuals pay a faking cost $c_{f}$ for each unit of change in their observable feature alone, $f(i,t+1)\cdot c_{f}$; and, finally, individuals pay a detection cost $c_{d}$ in the case of faking and effective detection, $d[f(i,t)]\cdot c_{d}$. Note that, if faking does not occur, the last term of the utility function is null, i.e., individuals expect to never pay any detection cost since *d*[*f*(*i*,*t*)]=0.

#### 2.3.2 Adaptation effort.

We keep track of individuals’ total adaptation effort and evaluate how this measure depends on social networks’ topology. The idea is to evaluate the average variation in the information regarding the real and observable features.

Let us define the **average improvement effort**, namely $AE_{I}$, by each user. For a given population of *N* individuals, let us define the average adaptation effort (improve) over the period (*t*,*t* + 1) as


$$\begin{array}{c} AE_{I}(t,t+1)=\frac{1}{N}\sum_{i=1}^{N}\Delta_{1}(i,t+1), \end{array}$$ (13)

with


$$\begin{array}{c} \Delta_{1}(i,t+1):=(x_{1}(i,t+1)-x_{1}(i,t)) \end{array}$$

as in Eq. (12), representing the variation in the information regarding the real feature *x*_1_ between time *t* + 1 and time *t* for a generic individual *i*.

Let us recall that all individuals in the population can decide whether to fake (by only modifying their observable feature *x*_2_), or truthfully improve (by modifying their real feature *x*_1_. We can thereby define the **average faking effort**, namely $AE_{F}$, as


$$\begin{array}{c} AE_{F}(t,t+1)=\frac{1}{N}\sum_{i=1}^{N}\Delta_{2}(i,t+1), \end{array}$$ (14)

with


$$\begin{array}{c} \Delta_{2}(i,t+1):=(x_{2}(i,t+1)-x_{2}(i,t)) \end{array}$$

representing the variation in the information regarding the observable feature *x*_2_ between time *t* + 1 and time *t* for a generic Individual *i*.

### 2.4 Behavioural imitation over networks

Let us introduce the possibility of imitation and assume that Individuals in the population can decide to imitate the behaviour of others Individuals in the population. Recall that adaptation consists in modifying feature values $\left(x_{1},x_{2}\right)$ and can thereby be formalized via an *adaptation vector* $\vec{u}(i)$, as


$$\begin{array}{c} \vec{u}(i)=(x_{1}(i,t+1)-x_{1}(i,t),x_{2}(i,t+1)-x_{2}(i,t))=(\Delta_{1}(i,t+1),\Delta_{2}(i,t+1)), \end{array}$$ (15)

taking as elements the variation in the information regarding both the real and observable feature, for each Individual *i*. Let us denote with:

- ${{\vec{u}}^{*}}_{m}(i)$ the vector resulting from utility maximization by Individual *i* (i.e., best response), based on utility function in Eq. (11), and
- $\overset{g}{\underset{P_{i}}{\underset{―}{\vec{u}}}}(i,net({𝒜}_{ij}))$ the vector resulting from the imitation of behaviour *g* of an observed pool $P_{i}$ of individuals in the population, for a given network topolo*g*y $net({𝒜}_{ij})$ identified via the type of network *net* and the specific adjacency matrix ${𝒜}_{ij}$. In particular, by introducing complex network effects, we assume that $P_{i}$ is the set of neighbors (nodes) directly connected to individual *i*, which may change depending on the network topology $net({𝒜}_{ij})$. The denser the network, the bigger is the size of $P_{i}$. Parameter *g* indicates whether Individual *i* imitates on the basis of the average value or maximum value, i.e., g∈{avg,max} – emulating real cases in which individuals use the information collected to imitate an average observed behaviour or an extreme observed behaviour.

The general imitation dynamics is formally introduced by assuming that individuals willing to imitate will adapt their behaviour by setting


$$\begin{array}{c} \vec{x}(i,t+1)=\vec{x}(i,t)+(1-\alpha )\cdot {{\vec{u}}^{*}}_{m}(i)+\alpha \cdot \overset{g}{\underset{P_{i}}{\underset{―}{\vec{u}}}}(i,net({𝒜}_{ij})), \end{array}$$ (16)

where parameter α∈[0,1] captures the imitation strength in the adaptation process. Fig 1d reports an illustrative example of the network structure and the imitation dynamics, meaning how each node can decide to act, i.e., based on i) best response, ii) imitation, highlighting α as imitation strength parameter. In this setting, limiting the set of imitators and individuals to be imitated to specific groups implies that, in a population, not everyone has the same visibility and likelihood to be influenced by others. This is directly connected to the complex network, its structure and topology. We shall also clarify that our model assumes a one-step imitation process and does not consider long-term dynamics of *information diffusion* (as in alternative works such as [31]).

#### 2.4.1 Networks and network topologies.

We model social networks where each Individual *i* can be seen as a node or vertex (Fig 1a). Let us assume *G* = (*V*,*E*) to be an undirected graph with *V* representing vertices and *E* edges. Let us denote with *N* = |*V*| the number of vertices, which correspond to the nodes for each Individual in our problem and *m* = |*E*| the number of edges in the graph. As indicator of the social network density, we consider the average degree ⟨k⟩=2m/N of the undirected network. Formally, each undirected network is also characterized by a specific adjacency matrix $A_{ij}$ and associated network topology $net(A_{ij})$ that we use to define the imitation process in Eq. (16).

**Fig 2:**
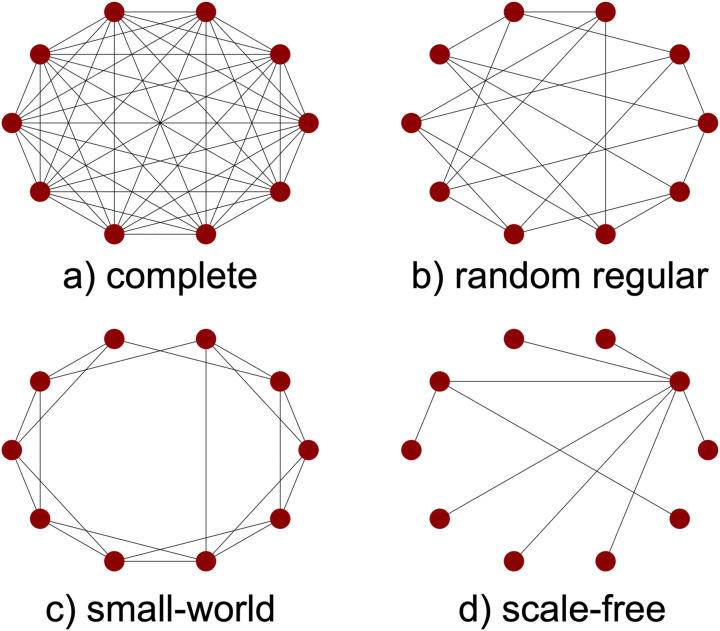
Complex networks. Illustrative example of the networks considered in this paper. Panel a) **complete graphs**, where all nodes are connected with each other. Panel b) **random-regular graph**, where all nodes are connected to the same number of neighbors, chosen randomly (i.e., all nodes have the same degree) [32]. Panel c) **small-world graphs**, characterized by high clustering coefficient and low average path length [33]; we consider a Watts-Strogatz network with *p* = 0.01 rewire probability. Panel d) **scale-free (Barabasi-Albert) graphs** [34] where nodes are connected through preferential attachment leading to heterogeneity in their degree distribution.

We consider different complex network topologies, capturing characteristics of individuals’ social networks. We test a set of alternative topologies to cover a wide range of networks, varying according to average degree, degree distribution and clustering coefficient. Fig 2 reports a visualization of the network structure for each type of topology considered in the paper: a) **complete graph**, in which all nodes are connected with each other; b) **random-regular graph**, in which all nodes are connected to the same number of neighbors [32], chosen randomly (i.e., all nodes have the same degree); c) **small-world graph**, characterized by high clustering coefficient and low average path length [33]; d) **scale-free (Barabasi-Albert) graph**, where nodes are connected through preferential attachment [34].

### 2.5 Experimental setup

We consider a population size of *N* = 1000 and thereby networks with *N* = 1000 nodes. At the beginning of each simulation (run) we sample the *x*_1_(*i*,0) and *x*_2_(*i*,0) of each Individual *i* from a probability distribution with support [0,1]. We assume an initial setting without faking, $x_{1}(i,0)=x_{2}(i,0)$, where *x*.(*i*,0) are distributed uniformly. In reality, individuals can misreport initially. However, the assumption $x_{1}=x_{2}$ at *t* = 0 is introduced to isolate the direct effect of stra*t*egic adaptation in a single step of classification. By assuming a setting where we start from $x_{1}=x_{2}$, we prevent False Positives or False Negatives before our strategic adaptation step. We repeat our simulations over 50 runs, starting from random initial conditions; the reported results are an average over these 50 runs. Furthermore, while in general we assign each initial feature independently, in Fig 8 we sort the sampled feature values and node degrees, assuming an assignment where the higher features values are assigned to the most connected nodes (higher degree). Each plot includes a shaded area showing the standard deviation across runs to reflect significance and sensitivity to initial conditions.

We first initialize the setting for agents, then consider the Institution classifying them. The classification problem at stake is a simple one, given the low number of dimensions in our feature space and the existence of a clear rule that determines individuals’ success (see Eqs. (1)-(2)). We perform classification with a linear SVM (Support Vector Machine), following the default configuration of scikit-learn [35].

After the classification of Individuals, we iterate over examples of *negative classification outcome* (i.e., rejection) so that these users may have a chance to adapt. We perform **synchronous** adaptation: first, we compute the best response adaptation vector for all agents (see ${{\vec{u}}^{*}}_{m}(i)$ in Eq. (16), Section 2.4). Afterwards, we compute the imitation vector for all agents, based on the best-response vector of their neighbors. Ultimately, these two vectors are combined (given the imitation strength α) following the dynamic in Eq. (16). After adaptation, we re-run the classification algorithm and compute the difference in the confusion matrix obtained (i.e., ΔFP, ΔTP, ΔFN, ΔTN). The S1 Appendix provides the pseudo-code underlying the experimental setup [29].

## 3 Results

In this section, we present and discuss the key results of our simulations focusing on the effects deriving from the main underlying drivers. Please recall that the overarching research question we address is: how will individuals adapt to increasingly transparent algorithms? The main conclusions arising from our results, detailed below, are:

- When individuals only engage in best response behaviour, transparent and actionable feedback can potentially raise the false positive rate, as agents may decide to strategically adjust their features to cross the decision boundary since full information is available (Section 3.1).
- Introducing imitation dynamics reveals a novel non-linear and non-monotonic effect: with moderate feedback noise, we can observe both overshooting and undershooting of the classification threshold when individuals implement their actions after algorithmic recourse, thus increasing false positives. This suggests a source of risk, since actionable recourse may prompt collective adjustments that push many individuals beyond the intended target region, ultimately harming classifier precision (Section 3.2).
- Feature-degree assortment impacts and amplifies the effects of imitation dynamic over complex networks. In particular, when assuming that features are correlated with network degree, this exacerbates the effects of social networks in strategic classification (Section 3.3).
- We monitor social costs of strategic adaptation by assessing the impacts of network degree on individuals’ adaptation effort. While imitation and social network effects could possibly influence both improving and faking behaviours, their impact is more pronounced in the case of faking (Section 3.4).
- From the perspective of the institution, the role of detection mechanisms and their effectiveness are crucial, since they are enablers to mitigate the risks of strategic classification on networks (Section 3.5).

### 3.1 Imitation and social networks affect the risks and benefits of algorithmic transparency

**Fig 3:**
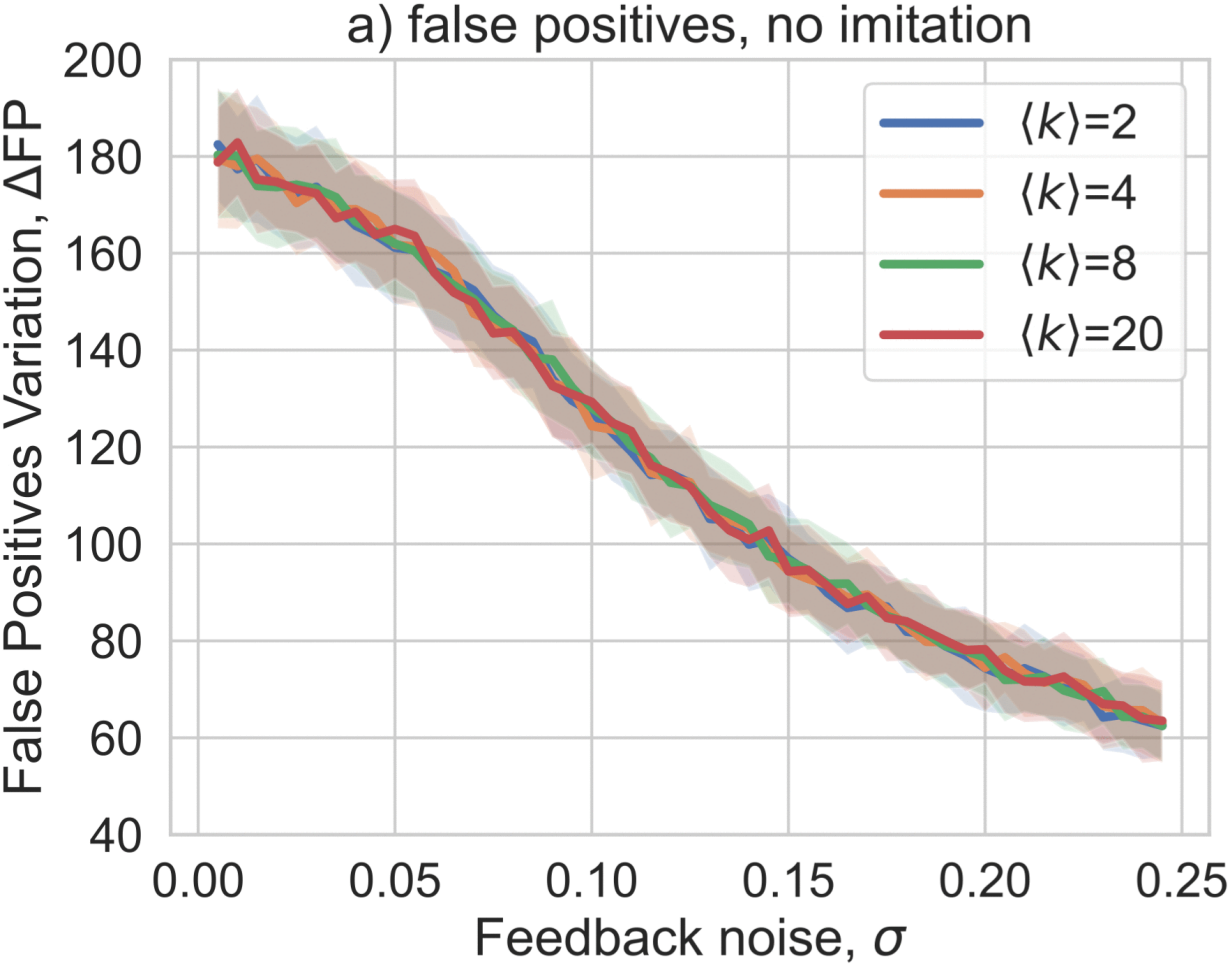
Network effects without imitation. The plot reports the difference in the number of False Positives after and before strategic adaptation (ΔFP on the *y*-axis) as a function of feedback noise (σ on the *x*-axis). We consider a population size of *N* = 1000 and thereby networks with *N* nodes. The reported results are an average over 50 runs starting from random conditions, with each individual starting with feature values $x_{1}(i,0)=x_{2}(i,0)$ sampled from a uniform distribution. The shaded area represents the standard deviation across independent runs. As reference, here we plot the variation in ΔFP when imitation is switched off, i.e., dynamic in Eq. (16) with α=0. Results are derived for the case of *Best response*, i.e., utility maximization. Network topology: Scale-free (Barabasi-Albert) graph, as in Fig 2d. Results are reported for different values of the network degree ⟨k⟩∈{2,4,8,20}. As expected, network properties, such as degree, have no impact in the observed results. Parameters: *b* = 1.0, $c_{\iota }=3.0$, ϵ=0, ϕ=0.5, γ=0.5, α=0.

Transparent feedback may impact strategic adaptation by Individuals. When the Institution provides exact information about the classification threshold θ used for the internal classification model (i.e., full transparency), there is a higher likelihood that Individuals decide to react by providing a new value of their observable feature *x*_2_(*i*,*t* + 1), higher than the real feature (*x*_1_(*i*,*t* + 1)). As a result, there is an increased likelihood in potential frauds, misreported information and data distribution shifts: in turn, it might lead to a high number of Individuals wrongly classified as positive. This has been shown in previous papers [28] and is also visible from Fig 3, where we observe that increasing σ leads to a decrease in ΔFP. This outcome results however from a setting where imitation is not considered (α=0) and all agents act based on best response.

**Fig 4:**
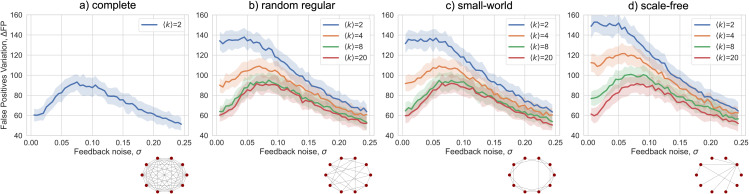
Transparency and accuracy of strategic classification on networks when imitating the average behaviour of neighbors. These plots report the difference in the number of False Positives after and before strategic adaptation (ΔFP on the *y*-axis) as a function of feedback noise (σ on the *x*-axis). We observe that increasing σ can increase ΔFP, translating in higher costs to the institution, meaning that an opaque decision threshold can negatively impact the institutions’ utility. This effect is more pronounced in networks with a higher degree. We consider a population size of *N* = 1000 and thereby networks with *N* nodes. The reported results are an average over 50 runs starting from random conditions, with each individual starting with feature values $x_{1}(i,0)=x_{2}(i,0)$ sampled from a uniform distribution. The shaded area represents the standard deviation across independent runs. For each plot, we consider a different network topology, following the examples in Fig 2 and as depicted in the small inset networks. For each plot, each curve is associated to a different value of average degree ⟨k⟩. Parameters: *b* = 1.0, $c_{\iota }=3.0$, ϵ=0, ϕ=0.5, γ=0.5. The imitation dynamic is defined in Eq. (16). For the complex network, results are based on an imitation strength α=0.3, assuming that Individuals imitate based on the average, i.e. g=avg. Results are reported for different values of the network degree ⟨k⟩.

When imitation plays a role in individuals’ adaptation (α=0.3), we observe that increasing the level of feedback noise σ can lead to a rise in the number of False Positives. Fig 4 reveals that for all social network topologies considered, and for high average degrees (⟨k⟩), initially increasing feedback noise σ leads to an increase in ΔFP. In such settings, transparency results in a relatively low risk of malicious adaptation. These results reveal that the potential risks and benefits of algorithmic transparency are also impacted by individuals’ behaviour imitation patterns and potential social network effects.

**Fig 5:**
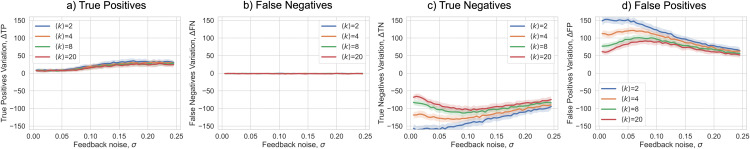
Transparency, Accuracy and Complex networks: metrics overview. The four plots report, respectively, from left to right, the difference in the number of True Positives, False Negatives, True Negatives and False Positives, after and before strategic adaptation (ΔTP, ΔFN, ΔTN, ΔFP on *y*-axis) as function of the level of feedback noise (σ on *x*-axis). We can observe that the changes in False Positives observed in the previous figures (and also panel d) is mainly due to a change in the number of True Negatives (panel **c**). This means that the effects of noise (σ) and network topology observed are mainly due to strategic adaptation by the users. Results are reported for different values of the network degree ⟨k⟩∈{2,4,8,20}. Parameters: *b* = 1.0, $c_{\iota }=3.0$, ϵ=0, ϕ=0.5, γ=0.5. The imitation dynamic is defined in Eq. (16). For the complex network, results are based on an imitation strength α=0.3, assuming that Individuals imitate based on the average, i.e. *g* = *avg*. Observe that ΔFP associated to this case is Panel d) in Fig 4 (same as the right plot in this figure). This refers to a scale-free (Barabasi-Albert) network topology (Fig 2d).

By assessing alternative metrics to ΔFP, we can observe that the variation in False Positives derives, essentially, from a transfer between True Negatives and False Positives (see Fig 5). The variation of False Negatives is negligible and the variation in True Positives has a slight increase only for the level of feedback noise above a certain value. This is explained by considering that in our simulation we assume an initial setting without faking, $x_{1}(i,0)=x_{2}(i,0)$. As a consequence, at time *t* = 0 we have zero False Positives and zero False Negatives. After adaptation, Individuals initially classified as True Negative are switching to either False Positives or True Positives.

**Fig 6:**
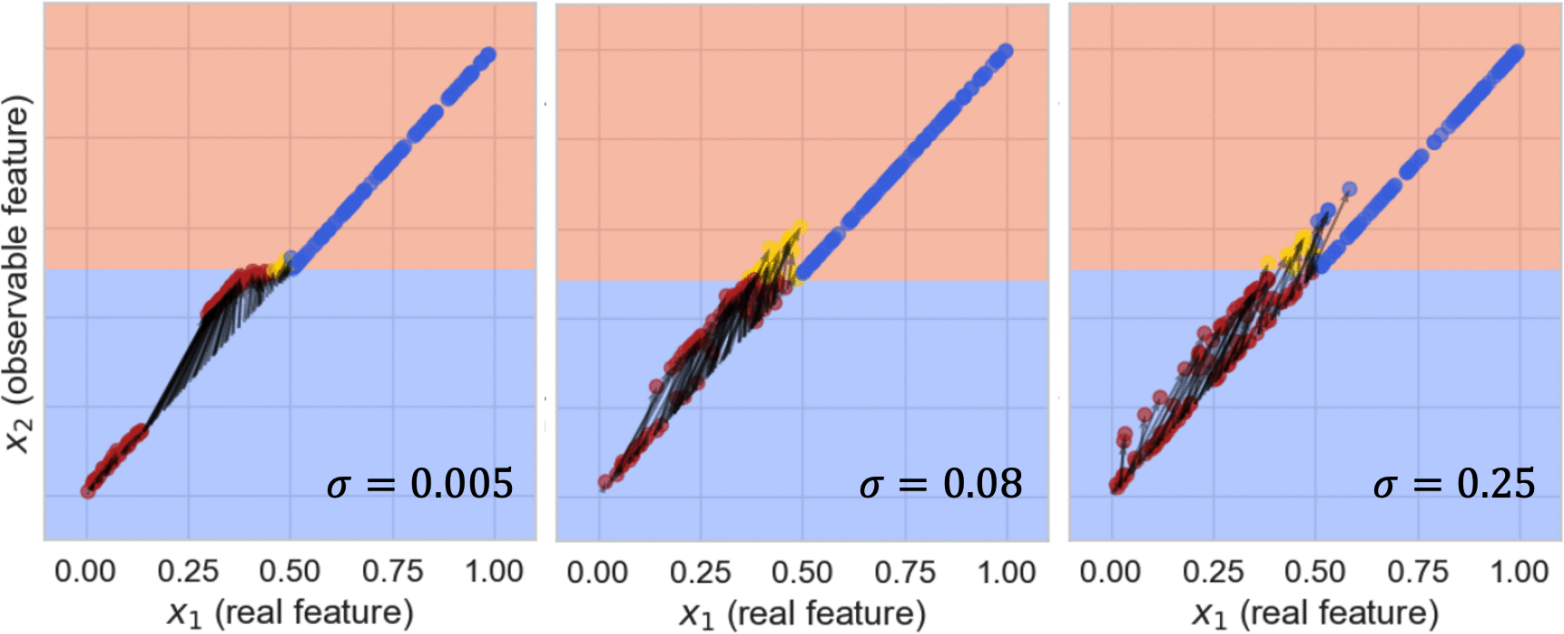
Intuition for the effect of noise on networks. Here we zoom-in on individuals’ adaptation for different levels of noise, when social networks are defined by a scale-free network (Barabasi-Albert, *N* = 200 and ⟨k⟩=20). Red circles represent True Negatives, after adaptation; yellow circles represent False Positives and blue circles represent True Positives. The leftmost plot corresponds to a setting where noise (σ) is low and ΔFP is also low, according to Fig 4. This occurs as imitating the average adaptation of a large pool of individuals in the population induces negative examples with a low real feature (*x*_1_) to undershoot the decision threshold even if increasing *x*_2_, thereby remaining classified as negative. The centre plot corresponds to a setting where noise (σ) is medium (σ=0.08) and ΔFP is also high; this occurs as noisy information about the institution’s decision rule induces individuals to adapt by overshooting the decision threshold; imitating average behaviour leads thereby to sufficient adaptation for negative examples to be classified as False Positives. Finally, the rightmost plot corresponds to a setting where noise (σ) is high (σ=0.25) and ΔFP is low; this happens as increased uncertainty about the decision threshold can induce some nodes to over-adapt and become True Positives.

Fig 6 provides an intuition for the impact of feedback noise on networks, when imitating based on average behaviour. Let us consider Individuals’ adaptation for different levels of feedback noise σ∈{0.005,0.08,0.25}, when social networks are defined by a scale-free network (Barabasi-Albert, *N* = 200 and ⟨k⟩=20). Red circles represent True Negatives, after adaptation; yellow circles represent False Positives and blue circles represent True Positives. Let us observe the plot on the left, for σ=0.005: feedback noise and ΔFP are low, according to Fig 4. This happens because imitating the average adaptation of a large pool of individuals in the population induces negative examples with a low value for the real feature (*x*_1_) to *undershoot* the decision threshold even when increasing *x*_2_, thereby remaining classified as negative. The plot in the middle reports the case of an intermediate value of the feedback noise σ=0.08 and ΔFP is high: noisy information about the Institution’s decision threshold θ induces Individuals to adapt by *overshooting* the decision threshold. In this scenario, imitating average behaviour leads thereby to sufficient adaptation for negative examples to be classified as False Positives. Finally, the plot on the right captures the case of σ=0.25, thus high feedback noise and low ΔFP: as uncertainty about the decision threshold increases, this can induce some nodes to over-adapt and become True Positives.

### 3.2 When imitating maximum behaviour, sparse networks reduce the risks of strategic classification

**Fig 7:**
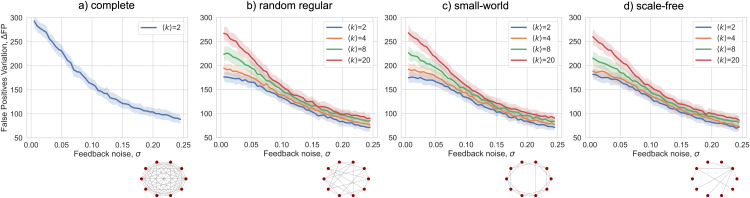
Strategic classification when imitating the most extreme behaviour of neighbors. These plots report the difference in the number of False Positives after and before strategic adaptation (ΔFP on the *y*-axis) as a function of feedback noise (σ on the *x*-axis). We consider a population size of *N* = 1000 and thereby networks with *N* nodes. The reported results are an average over 50 runs starting from random conditions, with each individual starting with feature values $x_{1}(i,0)=x_{2}(i,0)$ sampled from a uniform distribution. The shaded area represents the standard deviation across independent runs. For each plot, we consider a different network topology, following the examples in Fig 2 and as depicted in the small inset networks. For each plot, each curve is associated to a different value of average degree ⟨k⟩. Parameters: *b* = 1.0, $c_{\iota }=3.0$, ϵ=0, ϕ=0.5, γ=0.5. The imitation dynamic is defined in Eq. (16). For the complex network, results are based on an imitation strength α=0.3, assuming that Individuals imitate based on the most extreme behaviour, i.e. g=max. Results are reported for different values of the network degree ⟨k⟩. In this setting, feedback noise σ leads to a decrease in ΔFP, implying that uncertainty about the decision threshold reduces users’ gaming behaviour. We note however that also here networks play a role: the number of ΔFP is reduced in networks with lower average degree ⟨k⟩. For each plot, we consider a different network topology, following the examples in Fig 2 and as depicted in the small inset networks. Basically, this is the same configuration as in Fig 4 apart from the imitation behaviour, i.e. g=max here.

Next, we analyse how the previous results depend on individuals’ adaptation mode. In our model we assume that the social learning mechanism can be driven by the average g=avg or the max *g* = *max* feature observed in other individuals’. When *g* = *max*, we consider the role of imitating the most extreme behaviour. In Fig 7 we observe that, across social network topologies and network degrees, imitating maximum behaviour results in a negative correlation between feedback noise σ and ΔFP. We also observe that the risks of strategic classification in transparent settings are reduced in sparser social networks, i.e., with lower average degree.

### 3.3 Feature-degree assortment exacerbates the effects of social networks in strategic classification

**Fig 8:**
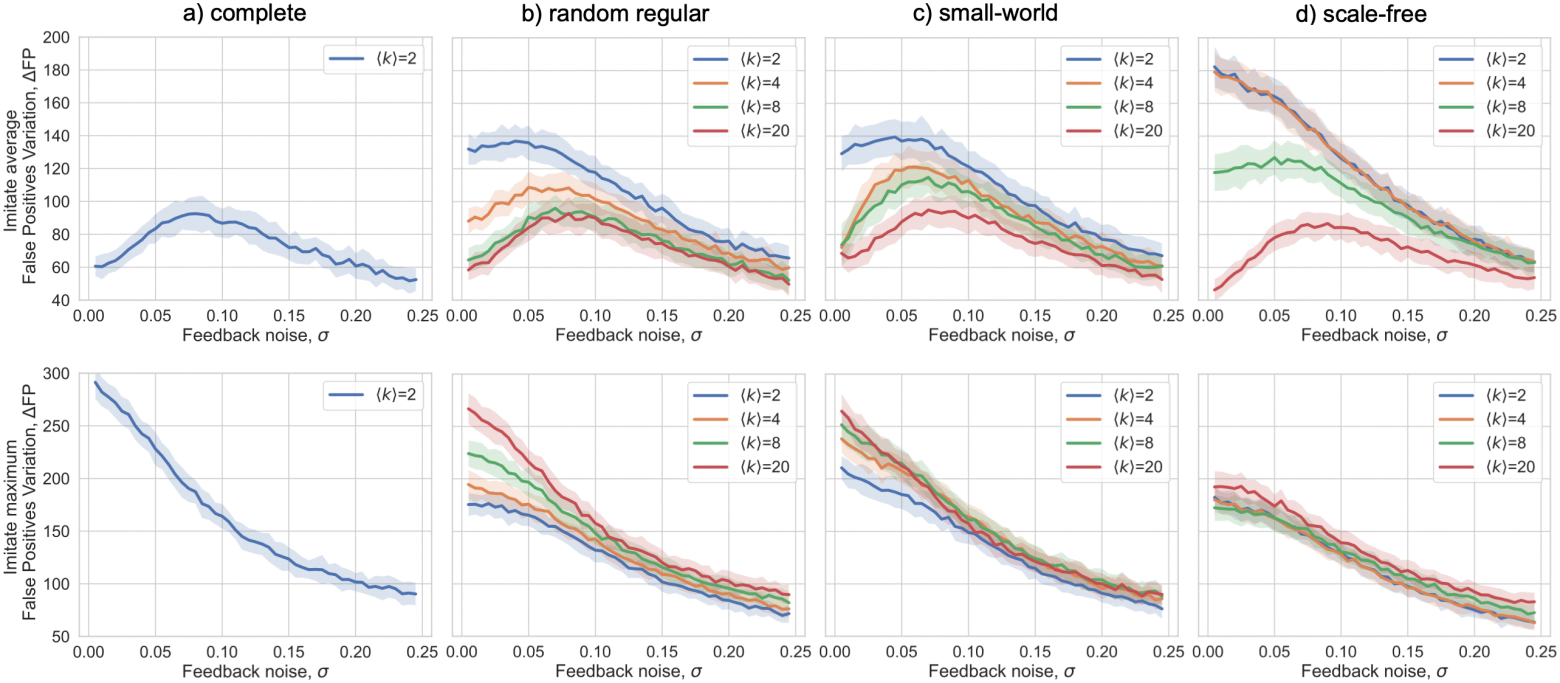
Results when features are sorted according to degree. These plots report the difference in the number of False Positives after and before strategic adaptation (ΔFP on the *y*-axis) as a function of feedback noise (σ on the *x*-axis). Same parameters and configuration as in Fig 4 for the plots at the top (Imitate average, *g* = *avg*); same parameters and configuration as in Fig 7 for the plots at the bottom (Imitate maximum, *g* = *max*). In reality, individuals’ network position could be correlated with their socioeconomic status [36]. Here we assume that features are correlated with network degree. We distribute features on networks assuming that the most connected node has the highest (initial) value of *x*_1_(*i*,0) and *x*_2_(*i*,0). We confirm the effect of noise (σ) and network topolo*g*y remains when there are degree-feature correlations in the networks. This can occur when highly connected nodes (i.e., with higher social capital) are also the individuals with more financial resources to re-pay a loan. We can observe that our previous results, discussed in Figs 4 and 7 are confirmed.

We have assumed that individuals’ features are distributed randomly in the networks. In reality, individuals’ network position could be correlated with their socioeconomic status [36]. In our subsequent experiments, we assume that features are correlated with network degree. We distribute features on networks assuming that the most connected node has the highest (initial) value of *x*_1_(*i*,0) and *x*_2_(*i*,0). Fig 8 presents the results in this setting. Our previous conclusions are also verified in this (more realistic) case.

**Fig 9:**
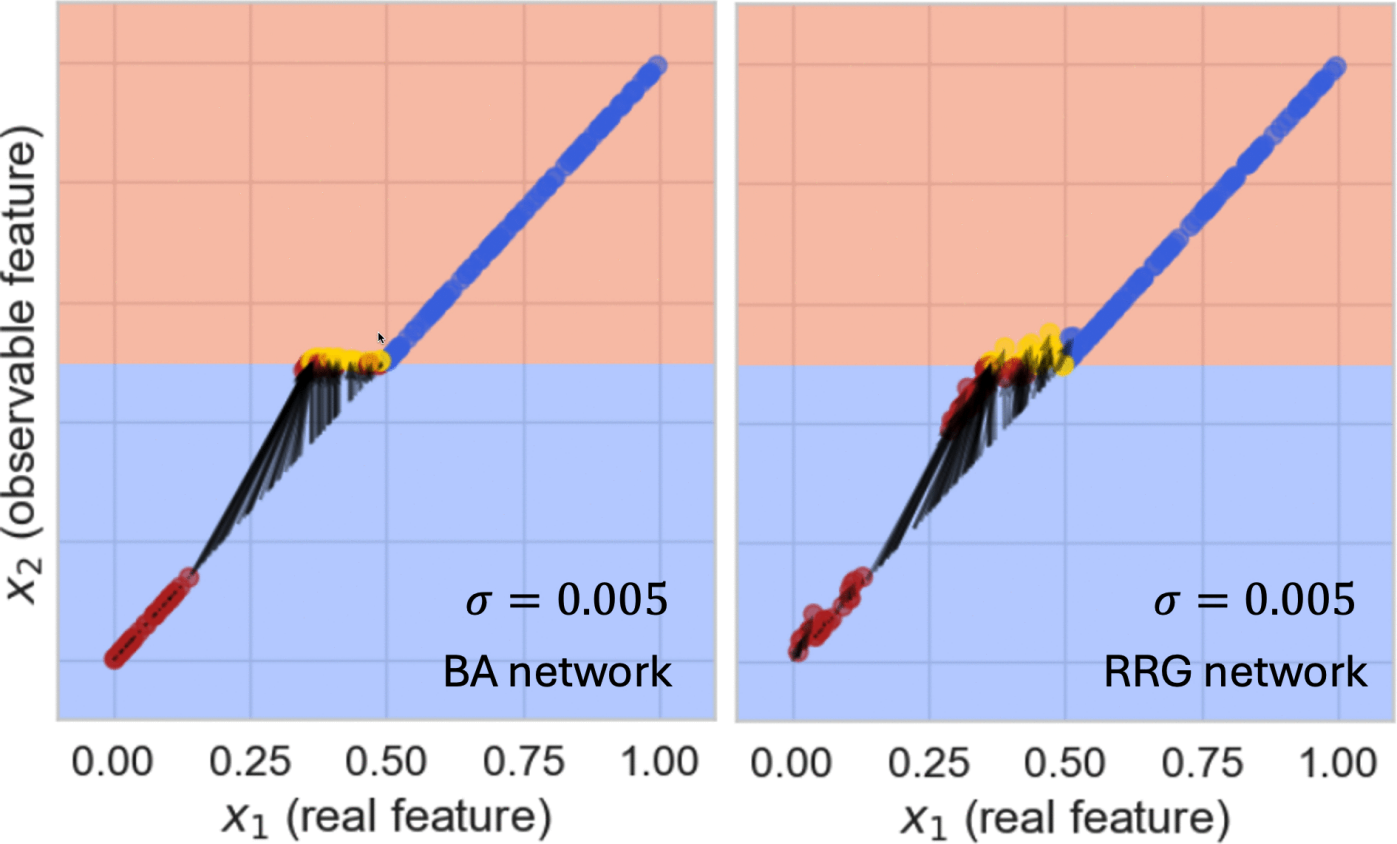
Intuition for the effect of network topology and degree-feature correlations. In a scale-free network (Barabasi-Albert *N* = 200, ⟨k⟩=4), nodes in highly connected positions have higher feature values (*x*_1_ and *x*_2_), thereby being classified as positive. As a result, they do not imitate and are not imitated by other nodes, which drastically reduces the number of links over which imitation can occur. In practice, this has a similar effect as reducing the networks’ average degree which, as observed in Fig 4, also leads to an increase in ΔFP in a setting of low noise (σ). Considering Random regular networks (right plot), where all nodes have the same degree, prevents feature-degree correlations, thereby reducing the effect of removing highly connected nodes from the pool of negative examples.

We note, however, that in this configuration, differences in the degree distribution of networks impact to a larger extent the observed results. The negative effects of feedback noise are more pronounced in scale-free networks, when users imitate average behaviour and networks have a higher average degree ⟨k⟩ (Section 2.4.1). Fig 9 provides an analysis to assess the impacts of both network topology and degree-features correlation. When considering the topology of a scale-free network (Barabasi-Albert *N* = 200, ⟨k⟩=4), we observe that nodes in highly connected positions have higher feature values (*x*_1_ and *x*_2_). Thus, they receive a favourable outcome by being classified as positive. As a result, based on our assumptions, they do not imitate and are not imitated by other nodes in the network: this effect drastically reduces the number of links over which imitation can occur. By interpreting this result, we can say that in practice, this has a similar effect as reducing the networks’ average degree which, as observed in Fig 4, also leads to an increase in ΔFP in a setting with low feedback noise (σ). Considering Random regular networks (right plot), where all nodes have the same degree, prevents feature-degree correlations, thereby reducing the effect of removing highly connected nodes from the pool of negative examples.

### 3.4 Network degree affects individuals’ adaptation effort

**Fig 10:**
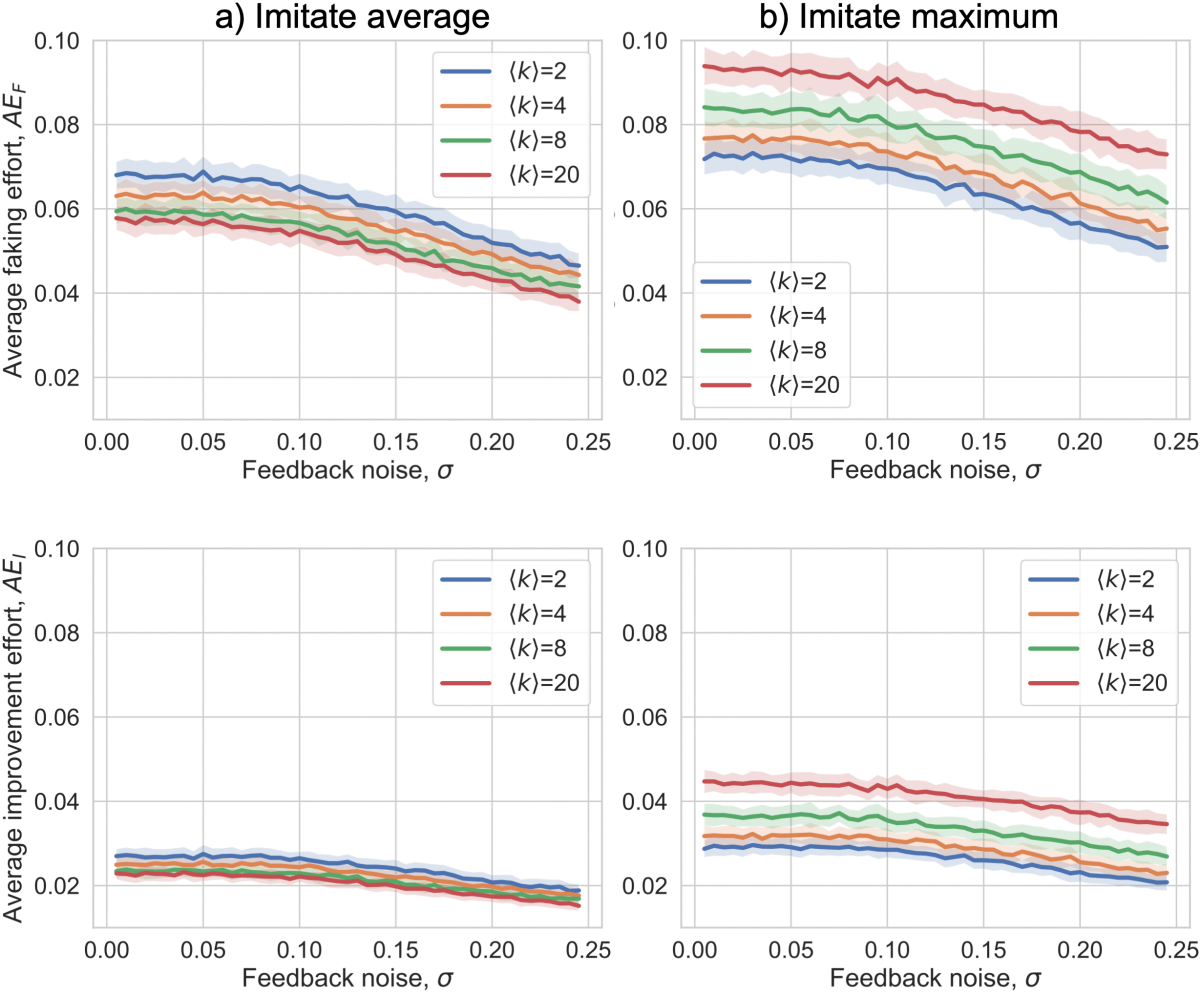
Adaptation effort. This figure reports the average adaptation effort of users ($AE_{F},AE_{I}$ on *y*-axis) for different levels of the feedback noise (σ on *x*-axis). We distinguish between *improvement effort* (bottom panels, $AE_{I}(t,t+1)$, defined in Eq. (13)) and *faking effort* (top panels, $AE_{F}(t,t+1)$, defined in Eq. (14)). We also consider adaptation based on imitation of average behaviour, i.e. *g* = *avg* (left panels) or imitation of the maximum behaviour, i.e. *g* = *max* (right panels). For each plot, we consider the same network topology, following one of the examples in Fig 2b, namely, a scale-free Barabasi-Albert network where nodes have on average ⟨k⟩ connections. We can observe that users’ chan*g*e their features to a large extent in networks with a smaller de*g*ree ⟨k⟩, when imitating the average behaviour. The opposite trend is observed when imitating the maximum behaviour. Moreover, we note that adaption decreases as noise σ increases and, overall, imitating maximum behaviour leads to a higher average adaptation. We consider a population size of *N* = 1000 and thereby networks with *N* nodes. The reported results are an average over 50 runs starting from random conditions, with each individual starting with feature values $x_{1}(i,0)=x_{2}(i,0)$ sampled from a uniform distribution. The shaded area represents the standard deviation across independent runs. For each plot, each curve is associated to a different value of average degree ⟨k⟩. Parameters: *b* = 1.0, $c_{\iota }=3.0$, ϵ=0, ϕ=0.5, γ=0.5. The imitation dynamic is defined in Eq. (16). For the complex network, results are based on an imitation strength α=0.3. Results are reported for different values of the network degree ⟨k⟩∈{2,4,8,20}. We use the same parameters as in: Fig 4b for Panel **a)** *Imitate average*, Fig 7b for Panel **b)** *Imitate maximum*.

Here we dive deeper into individuals adaptation process. Fig 10 reports the extent to which individuals adapt as function of the level of feedback noise (σ), highlighting the dynamics of the measures introduced. In particular, Fig 10 (bottom) reports the behaviour of the average adaptation effort to improve, i.e., $AE_{I}(t,t+1)$ in Eq. (13) and Fig 10 (top) reports the behaviour of the average adaptation effort to fake, i.e., $AE_{F}(t,t+1)$ in Eq. (14).

We observe that both $AE_{I}(t,t+1)$ and $AE_{F}(t,t+1)$ are decreasing w.r.t. the level of feedback noise σ. We also observe that the magnitude of adaptation is higher when considering imitation behaviour based on maximum *g* = *max*, in general, when compared with average *g* = *avg*.

Despite these similarities between honest ($AE_{I}(t,t+1)$) and malicious ($AE_{F}(t,t+1)$) adaptation, we observe important differences between these two adaptation modes. ⟨k⟩ We note that $AE_{I}(t,t+1)$ has a more limited range of variation compared to $AE_{F}(t,t+1)$. Moreover, $AE_{F}(t,t+1)$ is systematically higher than $AE_{I}(t,t+1)$ for all levels of feedback noise. This implies that, while imitation and social network effects could possibly influence both improving and faking, their impact is more pronounced in the case of faking. In this sense, as a result, faking behaviours during adaptation create an additional source of risk for institutions, since the negative effects could be further amplified. From a risk perspective, it is crucial to raise awareness about the potential burdens and risk scenarios, especially for the case of faking by imitating the maximum in a social network with high degree.

### 3.5 Effective detection mechanisms mitigate the risks of strategic classification on networks

**Fig 11:**
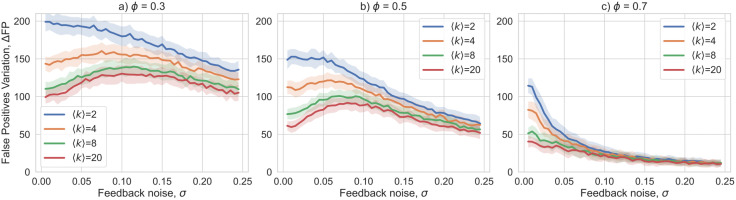
Strategic classification on complex networks under different levels of detection effectiveness. These plots report the difference in the number of False Positives after and before strategic adaptation (ΔFP on the *y*-axis) as a function of feedback noise (σ on the *x*-axis). We consider a population size of *N* = 1000 and thereby networks with *N* nodes. The reported results are an average over 50 runs starting from random conditions, with each individual starting with feature values $x_{1}(i,0)=x_{2}(i,0)$ sampled from a uniform distribution. The shaded area represents the standard deviation across independent runs. The plots assume a scale-free Barabasi-Albert network [34] as in Fig 2d. For the rest, we use the same configuration as Fig 4. For each plot, each curve is associated to a different value of average degree ⟨k⟩. Parameters: *b* = 1.0, $c_{\iota }=3.0$, ϵ=0, γ=0.5. The imitation dynamic is defined in Eq. (16). For the complex network, results are based on an imitation strength α=0.3, assuming that Individuals imitate based on the average, i.e. *g* = *avg*. Results are reported for different values of the network degree ⟨k⟩∈{2,4,8,20}. For each plot, we consider a specific value of the detection effectiveness (only variation w.r.t. Fig 4), left and right plot. We consider different detection probability factors (ϕ={0.3,0.5,0.7}). For higher levels of detection, the number of false positives decreases, overall. Observe that Fig 4d depicts the same results as Fig 11b.

First-party fraud includes intentionally misrepresenting intentions of financial situations and represents one of the main sources of fraud in digital transactions. With the present paper – besides allowing to study the role of networks in mitigating the risk of strategic adaptation – we account for the possibility that more effective faking detection methods are in place to mitigate this risk. It is crucial to ensure robustness of algorithmic design w.r.t. strategic manipulation and build strong detection mechanisms. In Fig 11 we observe that increasing detection effectiveness (ϕ) reduces the overall increase in the number of False Positives ΔFP. We consider simulations for different levels of detection effectiveness ϕ∈{0.3,0.5,0.7}. We observe that our previous conclusions are verified when detection effectiveness is relatively low (ϕ=0.3). For higher levels of detection effectiveness, the number of false positives decreases, overall (ϕ=0.7). In fact, as detection effectiveness increases, its impact dominates the ones deriving from networks, probably by creating more incentive to truthfully improve, regardless of the networks structure. This highlights the importance of having strong detection mechanism in place both for institutions and society, as this could contribute to mitigate the direct risks deriving from faking behaviours not based on real creditworthiness.

## 4 Conclusion

The increasing use of classification algorithms in high-stake domains requires attention to legal and ethical considerations. Algorithms should be transparent and trustworthy. Transparency and trustworthiness of algorithms are essential for sound, auditable and effective decision making. They enable independent scrutiny, reproducibility, and accountability. They help building meaningful explanations and actionable recourse for individuals affected by the decision. All in all, they contribute to support responsible adoption. However, when explanations and actionable recourse are provided, it is not always straightforward to anticipate how individuals will subsequently adapt. Individuals are embedded in complex social networks, and it is well-known that peer influence has a strong effect in decision making. In this paper we study the interplay between transparency, behavioural imitation and social network topology and their impacts on strategic classification.

By considering interactions among multiple stakeholders (Individuals and Institution) and Individuals’ social embedding (through imitation on complex networks), this work contributes to the scientific trend in *designing ethical multiagent systems taking into account their broader socio-technical context* [37,38]. The problem addressed in this paper is also related to the broader literature on Human-AI co-evolution, intended as *“the process in which humans and AI algorithms continuously influence each other,”* as mentioned in [39]. In particular, our paper introduces a general mathematical framework to study the interaction between individuals and one institution providing a service (i.e., credit lending) in the presence of social learning mechanism, via different structure of complex network topologies. This theoretical setting allows us to assess key aspects related to strategic classification and their long-term implications in terms of detection mechanisms, ethical AI and risk management.

Our results highlight that considering imitation over social networks results in a non-linear and non-monotonic relationship between transparency and algorithmic performance in most cases. In fact, we observe that increasingly opaque decision rules can negatively impact the utility of institutions, especially in dense social networks. We also observe that, when imitating the average behaviour among their connections, users change their features largely in networks with a lower degree. When we assume that network degree (i.e., number of connections) and the capacity to repay a loan are correlated, we observe that the number of false positives might further increase, as highly connected nodes are not imitated by other nodes, which drastically reduces the number of links over which imitation can occur. These results reveal that the potential risks and benefits of algorithmic transparency are also impacted by behavioural imitation patterns and social network effects.

Although our motivation and running example are related to banking and lending, the reported results extend to other domains. The need to develop algorithms robust to malicious adaptation has been reported in college admissions, standardized tests, hiring [40], recommender systems [41], spam detection [12] or even predictive policing [6]. A key assumption in our model is individuals’ willingness to exchange information with peers. In banking applications, this might occur in online fora or through private conversations, although this is a domain that naturally entails privacy concerns. In other settings, such as adaptation to standardized testing or hiring, individuals might have a different propensity to exchange information with peers.

Our simple model has limitations and can be improved in future works. On the one hand, we consider a simple two-dimensional feature space, to capture the difference between improving and imitation. Real settings are naturally more complex and involve feature spaces with higher dimensions. Furthermore, we focus on two imitation modes: imitate average behaviour and imitate the most extreme behaviour; we acknowledge that real peer pressure and behavioural imitation might deviate from these two modes as individuals might be influenced to a higher extent by specific peers. We assume that only individuals classified as negative imitate and are imitated. The reasoning is testing influence between *individuals that still need to adapt to be classified as positive*. In future work, however, it would be relevant to consider the role of information about neighbors that were previously classified as positive. We assume a one-step model, where individuals’ adaptation only occurs once. It would be pertinent to consider *the evolution of these dynamics over a longer time horizon*, by explicitly modelling the diffusion process of information in a social network beyond immediate neighbours. Finally, future work could consider models with increasing complexity at the individual and network level, by assuming individuals’ heterogeneity and partial information access, and network topologies stressing the role of homophily [42] and modularity [43]. Despite these limitations, our results provide already a robust *proof of concept* about the intricate effects of strategic classification in settings where behavioural imitation can occur over complex networks.

We finish by reflecting on the potential of our work to enable concrete advise and methods for practitioners. Let us take the example of credit lending discussed in this work as illustrative case study. Building on our modeling framework, institutions can consider creating synthetic datasets in which future data points are generated based on the principles of adaptation over social networks; evaluation teams can use this augmented dataset to monitor classifiers and identify early warnings for strategic classification risk.

Our methodological approach can also be translated into an implementable decision tools with clear assumptions, data needs, and actionable diagnostics, making the model’s value measurable, auditable, and deployable to support decision making and policy advisory for institutions. We believe this is a concrete step to further advance the discussion in the field of algorithms and human-AI co-evolution, from a strategic and modelling perspective.

## Supporting information

S1 Appendix Behavioural adaptation over complex networks: Simulation pseudo-code (PDF File [29]). (PDF)
