# Supplementary material for: The impact of transparency and imitation over complex networks in strategic classification
Source: PLoS One. 2026 Jul 10;21(7):e0346241. doi: 10.1371/journal.pone.0346241 (PMC13354108; doi:10.1371/journal.pone.0346241)
Supplement: S1 Appendix — (PDF) [file pone.0346241.s001.pdf]

The impact of transparency and imitation over complex networks in strategic classification

SUPPORTING INFORMATION

Flavia Barsotti[1,2 ‡*], Fernando P. Santos[3 *]

1 ING Analytics, ING Bank N.V., Amsterdam, The Netherlands

2 Delft Institute of Applied Mathematics, TU Delft, The Netherlands

3 Informatics Institute, University of Amsterdam, The Netherlands

‡ING Research Project Lead. The names of the authors are reported alphabetically.

* flavia.barsotti@ing.com, f.barsotti@tudelft.nl, f.p.santos@uva.nl

A Behavioural adaptation over complex networks: simulation pseudo-code

This Appendix provides the pseudo-code underlying the experimental setup. The code to replicate the results is available from the dedicated repository in [1].

The simulation procedure is based on a logic similar to the one in [2] and related supplementary information material [3]. In the present paper, the simulation procedure from [2] is generalized and extended to include: i) a parametric value for the threshold $\bar{\theta}$, instead of a fix value of 0.5; ii) the network topology component, i.e. captured by the adjacency matrix $\mathcal{A}_{ij}$; iii) the behavioural imitation type, i.e. $g \in \{avg, max\}$.

Algorithm 1 presents the main simulation cycle, where, at each run, individuals start from a random state at time $t = 0$ (truthful: $x_2(i,0) = x_1(i,0)$). After one step of classification, individuals can adapt their features at time $t = 1$:

Algorithm 1 Main simulation cycle, for a given *real* classification threshold ($\bar{\theta}$)

---

for $i \in N$ (Initialize all agents i in set N) **do**
    $x_1(i,0) \leftarrow x \sim U(0,1)$
    $x_2(i,0) \leftarrow x_1(i,0)$
    $y_i(0) \leftarrow \begin{cases} 1, & \text{with probability } \rho_i(x_1(i,0), \bar{\theta}) \text{ in Eq.(1):} \\ 0, & \text{otherwise} \end{cases}$
end for
Institution trains classifier (clf) using $x_2(i,0)$, $y_i(0)$
Classify all individuals using clf and compute metrics $FP(0)$, $TP(0)$, $FN(0)$, $TN(0)$
Assuming $\vec{x}(i,t) = [x_1(i,t), x_2(i,t)]$, imitation strength α, imitation behaviour based on $g \in \{avg, max\}$ and adjacency matrix $\mathcal{A}_{ij}$:
for $i \in N$ (Best response, Imitation on networks) **do**
    $\vec{x}(i,1) \leftarrow \vec{x}(i,0)+$ IMITATE(i, $\vec{x}(i,0)$, α, g, $\mathcal{A}_{ij}$)
end for
Classify all individuals using clf and $x_2(i,1)$.
Compute true label based on $x_1(i,1)$ and compute $FP(1)$, $TP(1)$, $FN(1)$, $TN(1)$
Compute
    $\Delta FP = FP(1) - FP(0)$
    $\Delta TP = TP(1) - TP(0)$
    $\Delta FN = FN(1) - FN(0)$
    $\Delta TN = TN(1) - TN(0)$

Algorithm 2 and **Algorithm 3** summarize adaptation, taking into account, respectively, adaptation through *Best response* (e.g. utility maximization) and adaptation based on *Imitation*. In this last case, individuals can decide to imitate based on average or maximum behavior, i.e. $g \in \{avg, max\}$ and taking into account the complex network structure via the adjacency matrix $\mathcal{A}_{ij}$. As **Algorithm 3** shows, we assume an imitation strength $\alpha \geq 0$ to weight the two components within the adaptation process.

Algorithm 2 Function used by individuals to update their own features after classification based on *Best response*, i.e. utility maximization, for a given pair (θ, σ)

---

function ADAPT(i, $\vec{x}(i,0)$, θ, σ)
    Input: individual id (i), and feature vector ($\vec{x}(i,0) = [x_1(i,0), x_2(i,0)]$), classification threshold θ, feedback noise σ
    Output: Adaptation displacement vector of individual i, $\vec{x}(i,1) - \vec{x}(i,0)$
Compute perceived threshold:
    $\hat{\theta}_i = \max(x_2(i,0)), N \sim (\theta, \sigma))$
Choose $\vec{x}(i,1) = [x_1(i,1), x_2(i,1)]$ maximizing Eq.(11):
    $x_1(i,1), x_2(i,1) \leftarrow \arg\max_{x_1(i,1), x_2(i,1)} u(i, t+1)$
return $(x_1(i,1) - x_1(i,0), x_2(i,1) - x_2(i,0))$
end function

Algorithm 3 Function used by individuals to update their own features after classification based on utility maximisation and imitation on complex networks. Imitation can be based on average behaviour or maximum behaviour of the neighbors, i.e. $g \in \{avg, max\}$.

function IMITATE(i, $\vec{x}(i,0)$, α, g, $\mathcal{A}_{ij}$)
Input: individual id (i), feature vector ($\vec{x}(i,0) = [x_1(i,0), x_2(i,0)]$), imitation strength α, type of imitation behaviour g, adjacency matrix $\mathcal{A}_{ij}$ for the complex network
Output: Adaptation displacement vector of individual i, $\vec{x}(i,1) - \vec{x}(i,0)$
Choose $x_1(i,1), x_2(i,1)$ maximizing Eq.(11):
    $\vec{u}^*_m(i) \leftarrow$ ADAPT($i, \vec{x}(i,0)$, θ, σ)
    $\underline{\vec{u}}^g_{P_i}(i, net(\mathcal{A}_{ij})) = [0, 0]$
    for $i \in N$ (Imitate average $g = avg$ or maximum $g = max$ displacement of other individuals in the network $net(\mathcal{A}_{ij})$, looking at neighbors P_i of individual i) **do**
        Identify the set of neighbors P_i for individual i
        $P_i \leftarrow (i, net(\mathcal{A}_{ij}))$
        for $j \in P_i$ **do**
            Vector of Best response for each individual j
            $\vec{n}^*_m(j) \leftarrow$ADAPT$(j, \vec{x}(j,0), \theta, \sigma)$
        end for
        if $g = avg$ **then**
            $imitAvg \leftarrow \sum(\vec{n}^*_{m(j)})/|P_i|$
            $imitNet \leftarrow imitAvg$
        else $g = max$
            $imitMax \leftarrow \max(\vec{n}^*_m(j))$
            $imitNet \leftarrow imitMax$
        end if
        $\underline{\vec{u}}^g_{P_i}(i, net(\mathcal{A}_{ij})) = \underline{\vec{u}}^g_{P_i}(i, net(\mathcal{A}_{ij})) + imitNet$
    end for
    return $(1-\alpha) \cdot \vec{u}^*_m(i) + \alpha \cdot \underline{\vec{u}}^g_{P_i}(i, net(\mathcal{A}_{ij}))$
end function

References

1. Barsotti F, Santos FP. The impact of transparency and imitation over complex networks in strategic classification (Supporting Information: Code Repository). PLOSOne. 2026. Available from: https://doi.org/10.17605/OSF.IO/NJUMG.

2. Barsotti F, Kocer RG, Santos FP. Transparency, Detection and Imitation in Strategic Classification. In: Raedt LD, editor. Proceedings of the Thirty-First International Joint Conference on Artificial Intelligence, IJCAI-22. International Joint Conferences on Artificial Intelligence Organization; 2022. p. 67-73. Main Track. Available from: https://doi.org/10.24963/ijcai.2022/10.

3. Barsotti F, Kocer RG, Santos FP. Transparency, Detection and Imitation in Strategic Classification (Supplementary Material). In: Proceedings of IJCAI-22; 2022. Available from: https://github.com/fp-santos/strategic-classification-imitation.
